# Calcification in Three Common Calcified Algae from Phuket, Thailand: Potential Relevance on Seawater Carbonate Chemistry and Link to Photosynthetic Process

**DOI:** 10.3390/plants10112537

**Published:** 2021-11-21

**Authors:** Pimchanok Buapet, Sutinee Sinutok

**Affiliations:** 1 Division of Biological Science, Faculty of Science, Prince of Songkla University, Hat Yai 90110, Songkhla, Thailand; pimchanok.b@psu.ac.th; 2 Coastal Oceanography and Climate Change Research Center, Prince of Songkla University, Hat Yai 90110, Songkhla, Thailand; 3 Faculty of Environmental Management, Prince of Songkla University, Hat Yai 90110, Songkhla, Thailand

**Keywords:** blue carbon, climate change, macroalgae, ecophysiology

## Abstract

Calcifying macroalgae contribute significantly to the structure and function of tropical marine ecosystems. Their calcification and photosynthetic processes are not well understood despite their critical role in marine carbon cycles and high vulnerability to environmental changes. This study aims to provide a better understanding of the macroalgal calcification process, focusing on its relevance concerning seawater carbonate chemistry and its relationship to photosynthesis in three dominant calcified macroalgae in Thailand, *Padina boryana*, *Halimeda macroloba* and *Halimeda opuntia*. Morphological and microstructural attributes of the three macroalgae were analyzed and subsequently linked to their calcification rates and responses to inhibition of photosynthesis. In the first experiment, seawater pH, total alkalinity and total dissolved inorganic carbon were measured after incubation of the macroalgae in the light and after equilibration of the seawater with air. Estimations of carbon uptake into photosynthesis and calcification and carbon release into air were obtained thereafter. Our results provide evidence that calcification of the three calcified macroalgae is a potential source of CO_2_, where calcification by *H. opuntia* and *H. macroloba* leads to a greater release of CO_2_ per biomass weight than *P. boryana*. Nevertheless, this capacity is expected to vary on a diurnal basis, as the second experiment indicates that calcification is highly coupled to photosynthetic activity. Lower pH as a result of inhibited photosynthesis under darkness imposes more negative effects on *H. opuntia* and *H. macroloba* than on *P. boryana*, implying that they are more sensitive to acidification. These effects were worsened when photosynthesis was inhibited by 3-(3,4-dichlorophenyl)-1,1-dimethylurea, highlighting the significance of photosynthetic electron transport-dependent processes. Our findings suggest that estimations of the amount of carbon stored in the vegetated marine ecosystems should account for macroalgal calcification as a potential carbon source while considering diurnal variations in photosynthesis and seawater pH in a natural setting.

## 1. Introduction

Since the mid-18th century, atmospheric CO_2_ has risen from 280 to 400 ppm, and is predicted to increase to 730–1200 ppm by 2100 as a result of human activities, such as the burning of fossil fuel, deforestation, agriculture and industrialization [1,2]. This has led to an increase in sea-surface temperature (ocean warming) and a decrease in ocean pH (ocean acidification), which have affected a range of marine floras and faunas [3,4,5]. Macroalgae play important roles as primary producers in the marine food chain and serve as habitat for various animals, which directly contribute to the livelihood and food security of the coastal community [6,7]. Recent studies have suggested that macroalgae may play a role in carbon sequestration, thus mitigating the changing climate, and offset anthropogenic carbon emissions to the atmosphere [8,9,10,11]. 

Calcifying macroalgae are intriguing organisms and have become a subject of interest for recent marine frontiers research [12,13,14,15] as they are a source of primary production via photosynthesis as well as CaCO_3_ production via calcification with relatively fast growth and turnover rates compared to corals [16,17]. However, their role in the carbon economy, whether they act as a net sink or source of carbon, remains a knowledge gap and debatable [18,19,20] mainly due to uncertainties about the fate of carbon associated with calcification. Previous studies suggest that in the pathway of calcification, the chemical reaction step modifies the carbonate chemistry of the seawater and releases CO_2_, resulting in a net loss of CO_2_ to the atmosphere [18,21,22,23]. A recent study by Kalokora et al. [20] found that calcification in a calcifying alga, *Corallina officinalis* L., can release a significant amount of CO_2_ to the atmosphere and become a source of CO_2_ if not refixed via photosynthesis in the system, highlighting the complex role of algal calcification on carbon capture potential in coastal areas [20].

Calcification is closely coupled to photosynthesis because photosynthetic carbon uptake decreases CO_2_, elevates pH and increases the CO_3_^2−^ proportion in the carbonate–bicarbonate system of the seawater, which promotes CaCO_3_ precipitation [24,25,26,27,28]. Respiratory CO_2_ release takes place in darkness, lowering seawater pH and promoting CaCO_3_ dissolution [13,29]. This relationship may change partly due to interspecific variations in the mechanisms and efficiency of photosynthetic carbon acquisition [30,31] and in the capacity to regulate the pH in the microenvironment [28,30,31,32]. A balance between all these simultaneous and interrelated processes partly shapes the daily and seasonal variations in calcification rates and carbon flux of shallow coastal water [26,33]. 

Species from genera *Padina* and *Halimeda* are calcifying macroalgae from divisions Phaeophyta and Chlorophyta commonly found in coastal subtropical and tropical habitats, including in Thailand [8,32,34,35]. While species from genus *Halimeda* have long been a model for calcification investigation, much less is known about species from genus *Padina. Padina* deposits aragonite crystals at cell surface, whereas *Halimeda* precipitates CaCO_3_ in the form of aragonite in intercellular spaces [36,37,38,39]. The source of CaCO_3_ for calcification of *Padina* is, thus, in the surface seawater layer on the thalli, whereas that of *Halimeda* is in the calcification fluid in interutricular spaces [36,37,38,39]. The species from genus *Padina* was categorized as lightly calcified where CaCO_3_ ranged from 9% to 63%, 21% and 38% of dry weight in *Padina pavonica*, *Padina japonica* and *Padina sanctae-crucis*, respectively [37,40,41], while the species from genus *Halimeda* has greater CaCO_3_ precipitation (81% and 86% of dry weight in *H. macroloba* and *H. cylindracea*, respectively [27]). Differential degrees of calcification and variation in microstructures are expected to be linked with their carbon use, particularly how they modify seawater chemistry and their carbon economy. In addition, the diversity of calcification sites and their proximity to pH-elevating processes (e.g., photosynthesis, H^+^ pumping and carbon concentrating mechanisms (CCMs)) relative to external seawater might affect their response to changing environments [14,28,39,42,43]. 

This study aims to provide a better understanding of the calcification process, focusing on its role in carbon economy and its relationship to photosynthesis in three dominant calcified macroalgae in upper sublittoral areas in Phuket, Thailand, *P. boryana*, *H. opuntia* and *H. macroloba* [44,45,46,47]. Our findings will improve estimations on the amount of carbon stored in the vegetated marine ecosystems and support the evidence-based management on mitigating the impact of human-induced climate change.

## 2. Results

**Figure 1:**
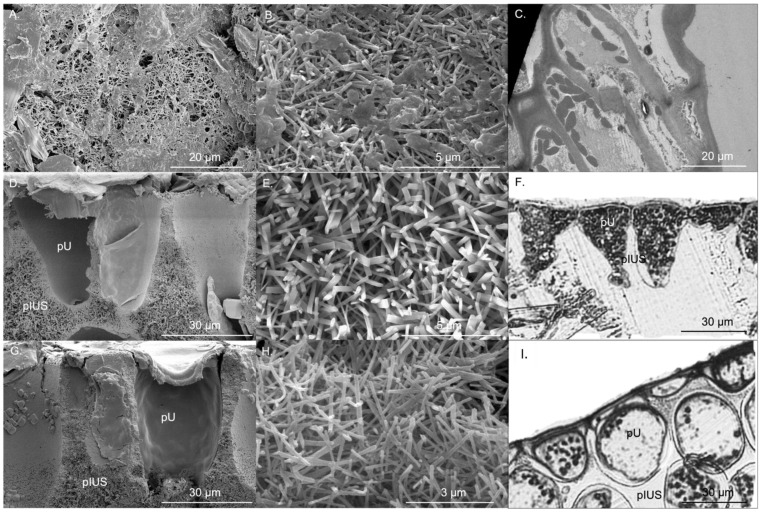
Aragonite crystal microstructure of *Padina boryana* (**A**,**B**), *Halimeda opuntia* (**D**,**E**) and *Halimeda macroloba* (**G**,**H**) from scanning electron microscope, cell of *P. boryana* (**C**), primary utricle (pU) and primary interutricle spaces (pIUS) of *H. opuntia* (**F**) and *H. macroloba* (**I**) from transmission electron microscope and compound microscope.

Specific thallus area (wet weight) of *P. boryana, H. opuntia* and *H. macroloba* were 37.09 ± 1.83, 6.41 ± 0.28 and 6.31 ± 0.40 cm^2^ g^−1^, respectively, while the specific thallus area (dry weight) of *P. boryana. H. opuntia* and *H. macroloba* were 163.88 ± 13.37, 14.84 ± 1.32 and 23.97 ± 1.50 cm^2^ g^−1^, respectively. Specific thallus area (wet and dry weight) of *P. boryana* was significantly greater than that of *H. opuntia*, and *H. macroloba* (Fisher’s LSD test, *p* < 0.001 and *p* < 0.001, respectively). CaCO_3_ in the form of aragonite of *P. boryana*, *H. opuntia* and *H. macroloba* were 46.89 ± 3.57, 81.44 ± 1.71 and 77.35% ± 1.44% dry weight, respectively, suggesting that *H. opuntia* and *H. macroloba* had greater CaCO_3_ precipitation than *P. boryana* (Fisher’s LSD test, *p* < 0.001 and *p* < 0.001, respectively), with no significant difference in %CaCO_3_ between *H. opuntia* and *H. macroloba* (*p* = 0.246). CaCO_3_ crystals of *P. boryana* were precipitated at the cell surface, whereas *H. opuntia* and *H. macroloba* precipitate CaCO_3_ in intercellular spaces. Figure 1 shows locations of primary utricles (pU), primary interutricle spaces (pIUS) and aragonite crystals of *H. opuntia* and *H. macroloba* and cellular structure and aragonite crystals of *P. boryana*. Aragonite crystals of *P. boryana, H. opuntia* and *H. macroloba* were needle- or rod-shaped and were 0.13 ± 0.02, 0.22 ± 0.02 and 0.12 ± 0.02 μm wide and 1.36 ± 0.05, 1.80 ± 0.09 and 1.24 ± 0.05 μm long, respectively (Figure 1). Significant longer and wider aragonite crystals were observed in *H. opuntia* (Fisher’s LSD test, *p* < 0.001 and *p* < 0.001, respectively). The CaCO_3_ crystal densities of *P. boryana, H. opuntia* and *H. macroloba* were 5.26 ± 0.41, 3.47 ± 0.12 and 5.65 ± 0.34 needles µm^−2^, respectively (Table 1). *H. opuntia* had significantly lower crystal density than *P. boryana* and *H. macroloba* (Fisher’s LSD test, *p* < 0.001).

The surface diameter of primary utricles (pU) of *H. macroloba* (28.31 ± 0.40 µm) was significantly larger than that of *H. opuntia* (23.67 ± 0.69 µm) (*t*-test, *p* < 0.001), while the diffusion pathway length of *H. macroloba* (22.11 ± 0.51 µm) was almost four-fold larger than that of *H. opuntia* (5.95 ± 0.25 µm) (*t*-test, *p* < 0.001) (Table 1).

**Figure 2:**
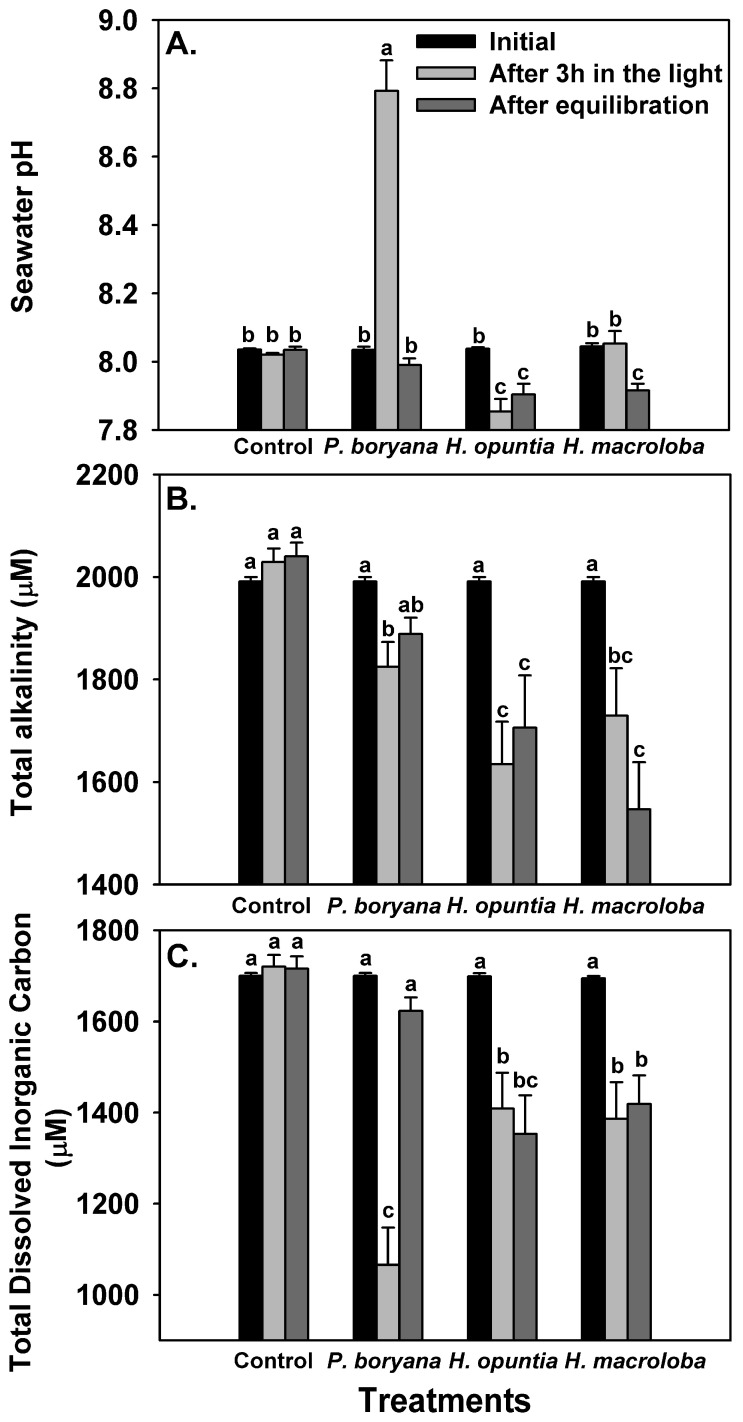
Changes in seawater chemistry: pH (**A**), total alkalinity (**B**) and total dissolved inorganic carbon (**C**) after incubation with *Padina boryana*, *Halimeda opuntia* and *Halimeda macroloba* under an irradiance of 150 μmol photons m^−2^ s^−1^ and after equilibration with air. The same set-up without macroalgae serves as the control. Bars without shared letters are considered statistically different (Fisher’s LSD test, *p* < 0.05). Data are means ± SE (n = 9).

The biological processes of the three macroalgae incubated in the light presented changes in seawater carbonate chemistry (Figure 2, Table 2). No significant alteration in controls (seawater without macroalgae) confirmed that the observed change in other treatments was associated with biological processes. Repeated-measures ANOVA showed that macroalgal species (*p* < 0.001), time of measurements (initial, after 3 h in the light and after equilibration with air, *p* < 0.001) and an interaction of the two factors (*p* < 0.001) significantly affected seawater pH, TA and total DIC (Figure 2). After exposure to the light for 3 h, a significant increase in seawater pH was detected in incubated *P. boryana* (reaching 8.79 ± 0.15, Fisher’s LSD test, *p* < 0.05), a reduction in seawater pH was detected in incubated *H. opuntia* (Fisher’s LSD test, *p* < 0.05) and no significant change in seawater pH compared to the initial values was observed in incubated *H. macroloba* (Figure 2A). Seawater pH was restored to the initial level after *P. boryana* was removed and the seawater was allowed to be equilibrated with air. In contrast, seawater pH remained low in treatment with *H. opuntia* (Fisher’s LSD test, *p* < 0.05 compared to the initial value) and reduced in treatment with *H. macroloba* (Fisher’s LSD test, *p* < 0.05). A significant decrease in TA was detected in the seawater incubated with all species tested after light exposure (Fisher’s LSD test, *p* < 0.05, Figure 2B). After equilibration, a recovery of TA was observed in treatment with *P. boryana*, whereas TA in the other two treatments remained low. Total DIC followed the same trend as observed in TA (Figure 2C). These shifts in pH and TA resulted in a change in CO_2_, HCO_3_^−^, CO_3_^2−^, saturation of calcite (ΩCa) and saturation of aragonite (ΩAr) (Table 2). Incubation of *P. boryana* in the light resulted in a decrease in CO_2_ and HCO_3_^−^ and an increase in CO_3_^2−^, ΩCa and ΩAr in seawater (Fisher’s LSD test, *p* < 0.05). After equilibration, CO_2_ and HCO_3_^−^ were restored to the initial levels, whereas CO_3_^2−^ ΩCa and ΩAr in the seawater became slightly lower than the initial values (Table 2, Fisher’s LSD test, *p* < 0.05). The incubation of *H. opuntia* and *H. macroloba* displayed similar responses. After light exposure, all parameters were reduced (Fisher’s LSD test, *p* < 0.05) except for CO_2_, which showed an increase in *H. opuntia* (Fisher’s LSD test, *p* < 0.05) and a slight decrease from the initial value, although not statistically significant in *H. macroloba.* Equilibration reinstated CO_2_ concentration but did not restore HCO_3_^−^ CO_3_^2−^ ΩCa and ΩAr (Table 2, Fisher’s LSD test, *p* < 0.05). The salinity and temperature of the seawater remained steady throughout the experiments (30 ppt; 30 °C–31 °C).

**Figure 3:**
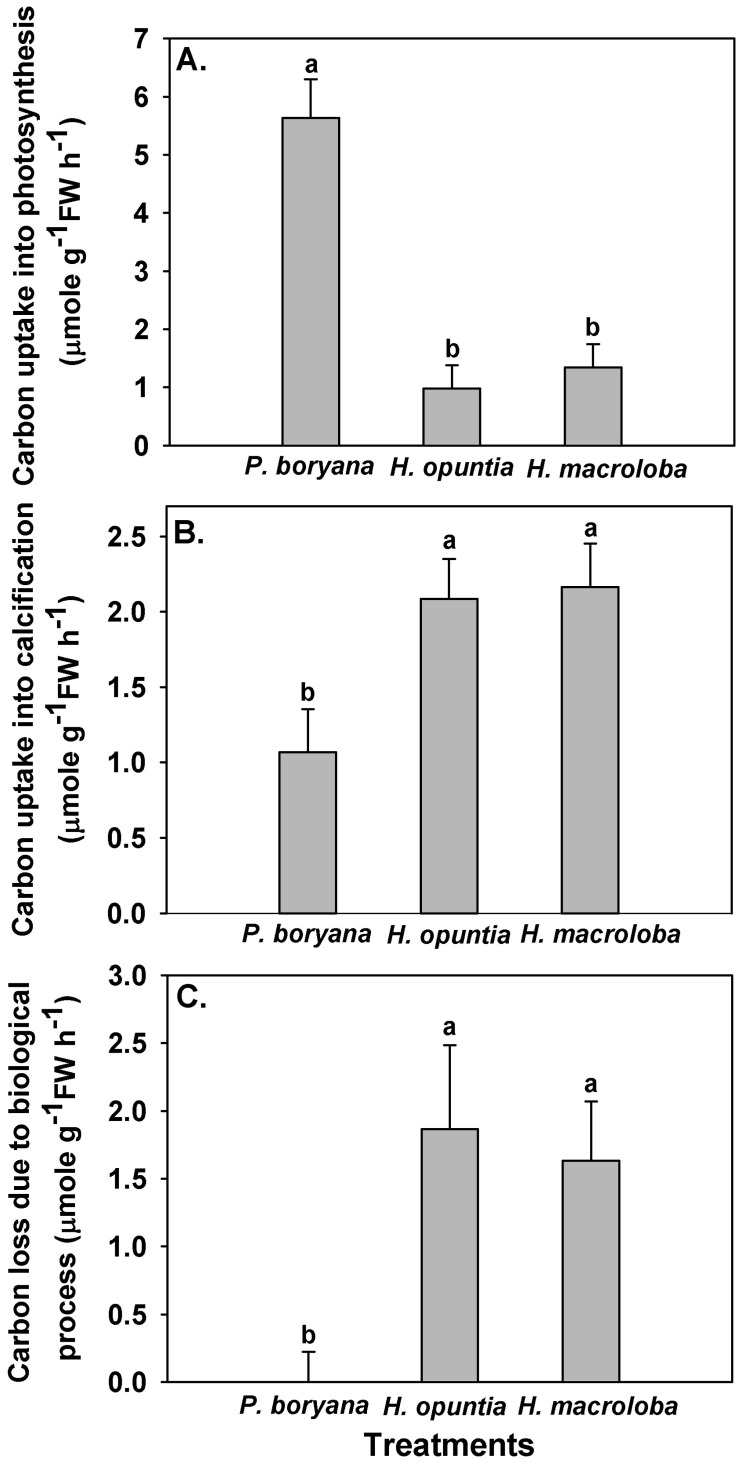
Carbon uptake by photosynthesis (**A**), carbon removal by calcification (**B**) and carbon lost to the air due to incubation (**C**) with *Padina boryana*, *Halimeda opuntia* and *Halimeda macroloba* under an irradiance of 150 μmol photons m^−2^ s^−1^. The fates of carbon were calculated according to Kalokora et al. [20]. Different letters above the bar indicate statistical difference (Fisher’s LSD test, *p* < 0.05). Data are means ± SE (n = 9).

Fates of the DIC associated with biological processes differed among species (Figure 3). Photosynthetic carbon uptake was highest in *P. boryana* (Fisher’s LSD test, *p* < 0.05), whereas *H. opuntia* and *H. macroloba* displayed comparable rates (Figure 3A), corroborating the pH drift and DIC results previously described in Figure 2 and Table 2. Opposite trends were observed in carbon removal by calcification and carbon loss due to biological processes (Figure 3B,C). The amount of carbon associated with both processes was lowest in *P. boryana* (Fisher’s LSD test, *p* < 0.05), whereas no difference was detected between *H. opuntia* and *H. macroloba*. With lightly calcified algae, *P. boryana* was more engaged in photosynthetic carbon uptake than in calcification, resulting in no net carbon loss. In contrast, two heavily calcified macroalgae removed a larger amount of carbon for calcification than for photosynthesis. The consequential carbon loss was almost as high as their carbon removal for calcification.

**Figure 4:**
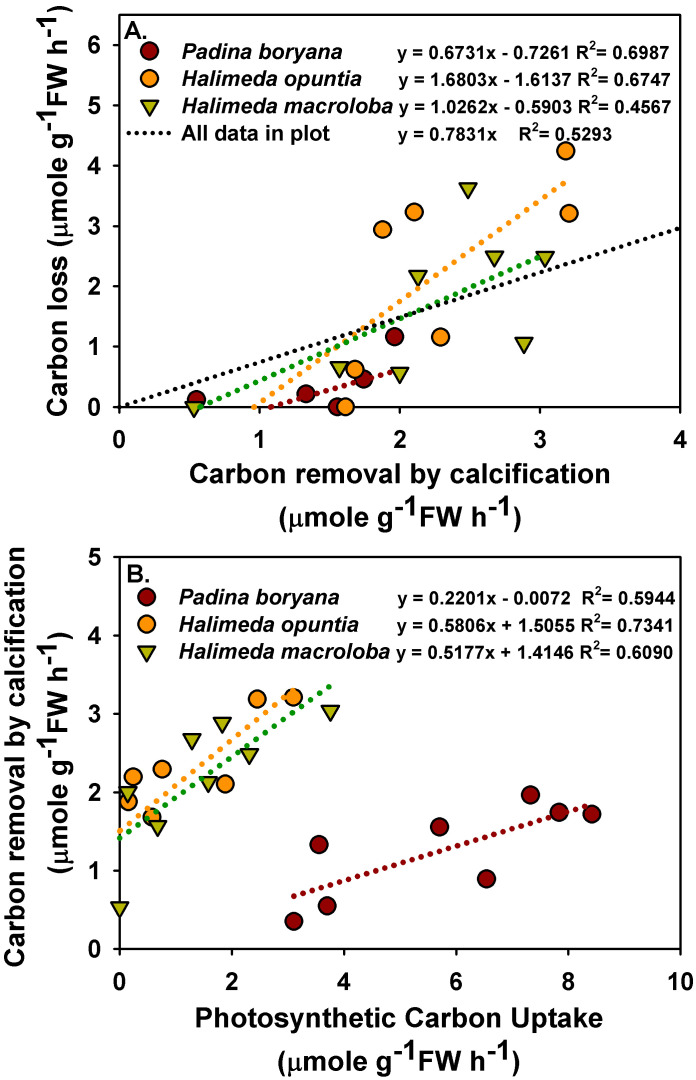
Relationship between carbon removal by calcification and carbon lost to air (**A**) and between photosynthetic carbon uptake and removal by calcification (**B**) in treatments incubated with *Padina boryana*, *Halimeda opuntia* and *Halimeda macroloba*. Fittings using linear regressions are shown as dotted lines, and the equations are displayed. Data are obtained from calculations shown in Figure 3.

There was a significant positive relationship between carbon removal by calcification and carbon loss to air in *P. boryana* and *H. opuntia* (Figure 4A, *p* = 0.005 in *P. boryana* and *p* = 0.0124 in *H. opuntia*), although both were not significantly related in *H. macroloba* (*p* = 0.0658). A trend line with an intercept = 0 was fitted using all the data points, and the ratio of carbon loss to air to carbon removal by calcification was 0.78. In addition, a significant positive relationship between photosynthetic carbon uptake and removal by calcification was detected in all species tested (Figure 4B, *p* = 0.0251, 0.0066 and 0.0223 in *P. boryana*, *H. opuntia* and *H. macroloba*, respectively). Photosynthetic carbon uptake caused 59.44%, 73.441% and 60.90% of the variation in carbon removal by calcification in *P. boryana*, *H. opuntia* and *H. macroloba*, respectively. 

**Figure 5:**
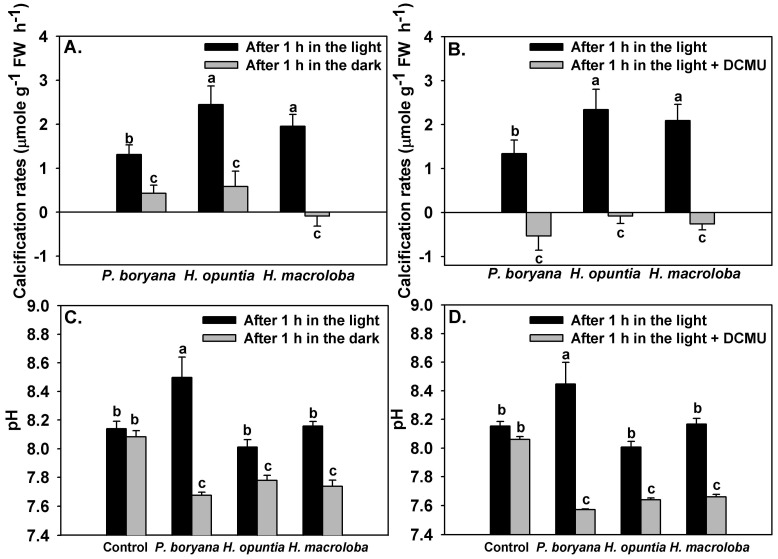
Effects of inhibition of photosynthesis on calcification rates of *Padina boryana*, *Halimeda opuntia* and *Halimeda macroloba* (**A**,**B**) and seawater pH (**C**,**D**) after 1 h incubation under an irradiance of 150 μmol photons m^−2^ s^−1^. Bars without shared letters are considered statistically different (Fisher’s LSD test, *p* < 0.05). Data are means ± SE (n = 9).

Inhibition of light reactions of photosynthesis affected the calcification rates of the three macroalgae and the pH drift of the seawater (Figure 5). Significant effects of incubation conditions (light and darkness) on calcification rates of the three macroalgae were detected (repeated-measures ANOVA, *p* < 0.001, Figure 5A). Similar to the results reported in Figure 3, calcification rates in *P. boryana* were lower than those in *H. opuntia* and *H. macroloba* (Fisher’s LSD test, *p* < 0.05). Calcification rates were lowered when macroalgae were incubated in darkness (Fisher’s LSD test, *p* < 0.05). *P. boryana* and *H. opuntia* maintained positive net calcification rates in darkness; however, net dissolution was detected in *H. macroloba*. Treatment with DCMU had a strong impact on the calcification process (repeated-measures ANOVA, *p* < 0.001) and induced dissolution in all species tested (Figure 5B). Seawater pH (Figure 5C,D) was affected by macroalgal species (repeated-measures ANOVA, *p* < 0.001), incubation conditions (repeated-measures ANOVA, *p* < 0.001) and interaction of the two factors (repeated-measures ANOVA, *p* < 0.001). Incubation conditions did not result in significant change in seawater pH of controls, indicating that changes observed in other treatments were associated with biological processes. An increase in seawater pH was detected after *P. boryana* was exposed to light for 1 h (Fisher’s LSD test, *p* < 0.05), whereas seawater from the incubation with *H. opuntia* and *H. macroloba* remained comparable to controls. Incubation in darkness resulted in a lower seawater pH as compared to incubation in the light in all treatments containing macroalgae (Fisher’s LSD test, *p* < 0.05, Figure 5C). Similar results were observed in the presence of DCMU (Figure 5D). 

## 3. Discussion

Our study showed that the biological processes of *P. boryana*, *H. opuntia* and *H. macroloba* significantly alter seawater chemistry but to a different extent, which is probably due to differences in photosynthetic carbon uptake and calcification. While both photosynthetic carbon fixation and calcification contributed to total DIC uptake in *P. boryana*, photosynthesis appears to be the main driver for a drastic decrease in DIC and a rise in seawater pH. In contrast, DIC uptake of *H. opuntia* and *H. macroloba* were largely associated with calcification and to a lesser extent, with photosynthesis, resulting in a change in pH with smaller magnitude. Higher calcification rates in *H. opuntia* and *H. macroloba* compared to *P. boryana* may also explain their higher accumulation of CaCO_3_ (1.6–1.7 times higher than in *P. boryana*). 

Both *Padina* and *Halimeda* were identified as active HCO_3_^−^ and CO_2_ users by means of CCMs [48]. Both can readily utilize HCO_3_^−^ for photosynthesis, although their actual mechanisms may differ depending on their modes of calcification [48,49]. Morphological characteristics and site of calcification influence calcification and photosynthetic process [39,50,51]. The sheet-like thallus of *P. boryana* has higher specific surface area than *H. opuntia* and *H. macroloba* thus they are expected to exhibit higher productivity and more efficient nutrient uptake for the same weight [52]. The morphology of primary utricle in *Halimeda* (e.g., size, orientation) and their lateral adhesion between adjacent primary utricles likely affect the diffusion pathway and ions uptake for CaCO_3_ precipitation (e.g., Ca^2−^, CO_3_^2−^) [39,50,51]. Diffusion pathways between primary utricles also control H^+^ fluxes from the bulk seawater into the primary interutricle spaces or removal of H^+^, a product of calcification, from the primary interutricle spaces to prevent H^+^ accumulation [39]. In addition, shorter diffusion pathway may lead to lower diffusive resistance for the influx of Ca^2+^ and potentially HCO_3_^−^ or CO_3_^2−^ from seawater to primary interutricle space, which potentially increases CaCO_3_ precipitation [39,50]. This suggests lower energetic cost to *H. opuntia* than *H. macroloba* in calcification [53,54,55], which might explain the higher CaCO_3_ content with larger crystal length and width and smaller crystal density in *H. opuntia* with a smaller surface diameter of the primary utricle and lower diffusion pathway length than *H. macroloba*. Our findings were also consistent with Peach et al. [39], which found strong inverse relationship between average CaCO_3_ content and primary utricle surface diameter. 

Variability in calcification rates in relation to photosynthesis among the three macroalgae corresponded well with reduced total alkalinity which did not recover after equilibration. In *H. opuntia* and *H. macroloba*, TA and DIC were decreased after incubation and remained low after equilibration, implying that CO_2_ loss from the seawater took place, which was consistent with a recent study in *Corallina officinalis* [20]. In contrast, with lightly calcified algae, *P. boryana* was more engaged in photosynthetic carbon uptake than in calcification, thus resulting in no net carbon loss. In this study, a positive correlation between carbon loss to air and calcification was presented, and the ratio of carbon loss to air to carbon removal by calcification was 0.78. This estimate is comparable to the values reported in *C. officinalis* (0.78; [20]) and slightly higher than those reported in the coral reefs (0.6; [56]). Notably, when each species was analyzed separately, different functional constants were obtained. These constants suggest that in order to obtain net carbon loss, a certain rate of calcification must be reached (negative y-intercept). While the results generally imply that macroalgal calcification may act as a source of CO_2_, the carbon economy of the coastal community is controlled by multiple processes. Kalokora et al. [20] suggested that the released CO_2_ may be refixed by a photosynthetic process, particularly of marine primary producers, such as seagrasses and other macrophytes. In Thai waters and other tropical habitats, calcified macroalgae, especially species from genus *Halimeda*, were often found coexisting with fleshy macroalgae and seagrasses [57,58,59]. Therefore, community photosynthesis may enhance calcification by increasing the pH and saturation state of CaCO_3_ in the surrounding seawater and simultaneously capture CO_2_ released from calcification during daytime [12,57]. 

There was a significant positive relationship between photosynthetic carbon uptake and carbon removal by calcification in all species tested, similar to previous studies in other calcifying algae [20,28,60,61,62]. Our findings provide supporting evidence that calcification and photosynthesis in *P. boryana*, *H. opuntia* and *H. macroloba* are closely coupled [24,28,32,63,64] but contradict the results of a study on the congeneric species, *Padina pavonica* and *Padina japonica*, which reported a lack of correlation between photosynthesis and carbonate deposition [40]. These discrepant results may be due to differences in calcification capacity, which was significantly higher in *P. boryana* in the present study [40]. This coupling between photosynthesis and calcification is mediated by a localized modification of seawater chemistry, generating a suitable microenvironment for photosynthesis and calcification. Calcium carbonate deposition of an individual promotes photosynthesis via an increase in proton extrusion, which subsequently increases CO_2_ availability readily for photosynthetic carbon uptake of the same individual [20,25,32,65]. Meanwhile, photosynthesis elevates the pH and saturation state of CaCO_3_ at the macroalgal thalli surface diffusive boundary layer and at the calcification site, thus promoting calcification [25,31,42,66,67]. Beer and Larkum [64] suggested that calcification in *Halimeda discoidea* is linked to pH in intercellular spaces controlled by the ratio of photosynthesis to respiration. Recent studies have revealed that higher pH at the diffusive boundary layer in the light in many tropical calcified macroalgae, including *Halimeda*, is maintained by photosynthetic CCM involving a HCO_3_^−^–H^+^ symport, OH^−^ antiport and HCO_3_^−^ dehydration catalyzed by external carbonic anhydrase [14,28]. Our study showed that approximately 4.5–4.7 mols of carbon were taken in photosynthesis for every mol carbon fixed into calcification in *P. boryana*. While regressions in *H. opuntia* and *H. macroloba* did not show the x and y intercepts of zero, approximately 0.5–1.0 and 0.5–1.2 mols of carbon were taken in photosynthesis for every mol carbon fixed into calcification in *H. opuntia* and *H. macroloba*, respectively. Different ratio numbers were reported in previous studies: 4–8 for *Halimeda* [68], 1.6 for four deep-water *Halimeda* [63] and 1 and 1.5 for *C. officinalis*, respectively [20,60], indicating that this is a variable depending on local adaptation and not only species-specific.

The dependency of calcification on photosynthesis was further illustrated in the subsequent experiment in which both light-dependent and photosystem II (PSII) electron transport-dependent calcification was observed. Incubation in darkness significantly reduced calcification rates in all tested species and simultaneously reduced seawater pH. As *H. opuntia* and *H. macroloba* deposit aragonite crystals in the intercellular space between utricles, which is semi-separated from external seawater, these algae may be more resistant to the pH change in surrounding water than *P. boryana*, which precipitate aragonite crystals externally at the thallus surface [40,68,69]. However, our results indicate that *P. boryana* was the least pH-sensitive, sustaining the highest percentage of calcification rate relative to the rates in the light (approx. 33%) at the lowest final pH (7.67 ± 0.02). This is evident as *Padina* is among the persistent macroalgal genus in acidified environments [38,41,70,71,72,73,74]. Meanwhile, *H. macroloba* was the most sensitive species, displaying net dissolution after 1 h of darkness at the highest pH (7.74 ± 0.04) among all species. Minimal calcification and dissolution were also observed in species from genus *Halimeda* in the field condition at nighttime [13,14,75]. Differences in sensitivity to pH between *H. opuntia* and *H. macroloba* may be due to different morphological characteristics, such as branching and crystal microstructure [76]. While multitudinous branches forming tight complex structures in *H. opuntia* may generate greater diffusive boundary layer thickness and consequently allowing better regulation of their microenvironment as previously observed in coralline algae [77], shorter and thinner crystals found in *H. macroloba* may be more susceptible to dissolution and may require more crystal nucleation, which is a major rate-limiting step for precipitation of CaCO_3_ [78]. In addition, inhibiting PSII electron transport using DCMU imposed stronger negative effects on calcification of all tested species, with *P. boryana* as the most affected. This emphasizes that certain mechanisms associated with calcification are species specific, and the processes and factors associated with functioning photosynthetic electron transport are more important facilitators of calcification than other light-dependent but PSII-independent processes, such as light-triggered H^+^ transport pumps [28,31,64]. Nevertheless, more adverse effects of DCMU could be attributed to a lower pH in the seawater, promoting dissolution in all species [13,38]. Comparisons among different species of calcified macroalgae in our study suggest that CaCO_3_ precipitation is linked to photosynthesis and pH, and may vary depending on the morphological features. However, these interrelations remain unclear and require further investigations.

Our results provide experimental evidence that calcification of the three common calcified macroalgae in Phuket, Thailand is a potential source of CO_2_, supporting a recent study on *C. officinalis* [20]. Calcification by *H. opuntia* and *H. macroloba* lead to greater loss in CO_2_ per biomass weight than *P. boryana*. Nevertheless, this capacity varies on a diurnal basis, as calcification is highly coupled to photosynthetic activity. Our results also imply that future acidification will impose more negative effects on *H. opuntia* and *H. macroloba* than on *Padina boryana*, and the effects on calcified macroalgae may be worsened in darkness. Therefore, further investigations on the carbon budget of the coastal ecosystem should account for calcification as a potential carbon source while considering diurnal variations and the buffering capacity of the primary production of marine vegetation in the natural setting [12,26,57].

## 4. Materials and Methods

### 4.1. Sampling and Acclimatization

*P. boryana*, *H. opuntia* and *H. macroloba* were carefully collected with their holdfasts intact from Tung Khen Bay, Phuket (7.810057; 98.404100), where all species were found in high densities and growing on calcareous sediment with depth between 0.5 and 1.0 m. As our investigations were solely focused on the populations growing in the upper sublittoral areas, differences due to varying depth and irradiance were not considered. Cleaned samples were transported to the aquaria facility of Coastal Oceanography and Climate Change Research Center, Prince of Songkla University, in cool boxes containing seawater collected from the sampling site. Additional seawater was collected in sealed dark polypropylene bottles and fixed when necessary. Seawater pH, salinity and total alkalinity (TA) were immediately determined upon arrival at the laboratory (pH = 8.1, salinity = 30 and TA = 2.04 ± 0.05 mM). 

At our aquaria facility, the samples were rinsed to remove the remaining sediment and epiphytes. Collected samples were maintained in aquaria filled with sterile seawater (pH = 8.1, salinity = 30 PSU and TA = 1.90 ± 0.08 mM) with constant aeration under ambient temperature (27 °C–32 °C). The irradiance of approximately 150 μmol photons m^−2^ s^−1^ was provided with aquarium LED light (A601, Chihiros, Cixi, Zhejiang, China) with a 12:12 h light–dark cycle. An irradiance of 150 μmol photons m^−2^ s^−1^ (measured using LI-250A light meter; LICOR, Germany) was chosen based on the minimal saturating irradiance (E_k_) derived from the rapid light curves (RLC; Ralph and Gademann [79]) conducted using the collected samples (data not shown). Throughout the acclimation period, pH, salinity and TA were monitored regularly to avoid changes due to evaporation, and distilled water was added as necessary. Prior to the experiment, the health of the macroalgae was evaluated by a pulse amplitude modulated fluorometer (Diving-PAM, Walz, Effeltrich, Germany) using the maximum quantum yield (F_v_/F_m_) as a biomarker. Subsequent experiments were carried out with algal thalli with an F_v_/F_m_ ≥ 0.7. Experiments were conducted within 7 days of the collection date after which the samples were discarded and fresh materials were collected. Each experimental run consists of all species and all treatments. The run was repeated with new biological materials.

### 4.2. Characterization of Calcification and Its Effect on Seawater Chemistry

#### 4.2.1. Experimental Design

Each macroalga was weighed to reach the wet weight of approximately 5 g and placed in 250 mL flasks containing a magnetic bar (0.4 × 1.0 cm in size) and filled with 250 mL of seawater. The flasks were tightly closed with rubber stoppers and securely wrapped with parafilm. Flasks filled with seawater without macroalgae were used as controls and were included in the experiment. The flasks were arranged on top of magnetic stirrers where mixing occurs and the effect of the diffusive boundary layer was minimized. The assembly of the experimental set-up was done in dimmed light. Subsequently, an irradiance of approximately 150 μmol photons m^−2^ s^−1^ was provided by aquarium LED light (A601, Chihiros, China) for 3 h. The light source was positioned on the side so that the macroalgae were not shaded by the stoppers. When incubation in the light was finished, the macroalgae were removed from the flasks. The remaining seawater without macroalgae was then allowed to be equilibrated with air using a compressed air pump for an additional 4 h (the minimum duration for the seawater to reach stable pH, predetermined from the preliminary results). The macroalgae from each flask were rinsed and dried using a paper towel and kept at −20 °C for further assessment of the surface area, CaCO_3_ accumulation and microstructural analyses (See below).

Parameters related to seawater chemistry, namely seawater pH, TA and salinity, were measured at three steps: (1) the initial step before light exposure, (2) after 3 h of light exposure and (3) after equilibration with air. Seawater temperature was also recorded. These parameters were used to calculate total dissolved inorganic carbon (DIC), calcification rates, photosynthetic carbon uptake and carbon loss to air, further detailed in Section 4.2.3. Measurements for each species and each treatment were based on nine biological replicates (n = 9). 

#### 4.2.2. Determination of Surface Area, CaCO_3_ Accumulation and Microstructural Analyses

Thalli of *P. boryana*, *H. macroloba* and *H. opuntia* were cut into parts (individual lobe for *P. boryana* and individual segment for *H. macroloba* and *H. opuntia*) and spread on a surface lined with rulers, and the image of each sample was taken. The surface area was determined using image processing software (ImageJ V. 1.53e, Rasband W.S., U.S. National Institute of Health, Bethesda, Maryland, USA) from images of samples [80,81].

Samples were rinsed with distilled water and dried until constant weight at 60 °C. The CaCO_3_ accumulation of each sample was investigated by dissolving thalli in 5% HCl for 1 h and measuring the dry weights before and after CaCO_3_ dissolution using an electronic balance [27]. 

Images of aragonite CaCO_3_ crystals were examined using a scanning electron microscope (SEM Apreo, FEI, Czech Republic) at the Office of Scientific Instrument and Testing, Prince of Songkla University. The instrument was operated at 20 kV w in high vacuum mode and imaged using the secondary in-lens detector. Samples were mounted on aluminum stubs and then placed in a gold coating unit (SPI Supplies, USA) operated at a 40 mm working distance. Length, width and density of aragonite crystals (n = 4–6) were determined using image processing software. Aragonite crystals with complete needle structure at the top layer in the images were chosen. Ten crystals per replicate were used to calculate average size following the method described by Sinutok et al. [35].

Thalli of *P. boryana* and apical segments of *Halimeda* were fixed in 2% glutaraldehyde in 0.05 M phosphate buffered saline and stored at 4 °C. Fixed samples were posted-fixed in 1% OsO4 buffer, and dehydrated through a series of ethanol concentrations (70%, 80%, 90% and 100%). Samples embedded in epoxy resin and were cut into thin sections (80 nm) using a microtome (MT XL microtome, RMC, USA) and stained with 2% uranyl acetate (3 min). Stained sections of *P. boryana* were examined with a transmission electron microscope at 160 kV (JOEL, JEM-2010, Japan), while stained sections of *H. opuntia* and *H. macroloba* were examined using a compound microscope (DM500, Leica Microsystems, Germany). Surface diameter of primary utricle (pU) and diffusion pathway length were determined using image processing software as previously described.

#### 4.2.3. Determination of Carbonate Chemistry-Related Parameters and Fates of Carbon Related to Biological Processes 

To avoid creating large headspace in incubation flasks, parameters related to seawater chemistry at the initial step were determined from the samplings drawn from the prepared seawater for the experiment and not directly from the flasks. After light exposure and after equilibration, seawater was collected from each flask using a 50 mL syringe with an extended rubber tube. Total alkalinity was determined using a total alkalinity mini-titrator for water analysis (HI-84531-02, Hanna Instruments, Woonsocket, RI, USA) according to the manufacturer’s instruction. Salinity was measured using an optical refractometer, and seawater pH was measured using a bench-top pH meter (Ohaus RL150, Russell, USA) according to the manufacturer’s instructions. 

The measured seawater chemistry parameters were later used to calculate DIC, its distribution among different forms (CO_2_, HCO_3_^−^ and CO_3_^2−^) and saturation state of aragonite (ΩAr) and calcite (ΩCa). Calculation was performed using the software CO_2_ sys.xls [82]. 

Calcification rates were calculated following alkalinity anomaly protocol (as described in [20]), whereby a decrease in TA by two equivalents when one mole of CaCO_3_ is precipitated [83,84]. Calcification rates were normalized against macroalgal wet weight. 

In addition to calcification rates, the difference in DIC and TA in seawater collected at the initial time point after incubation in the light and after equilibration can estimate photosynthetic carbon uptake and carbon loss from the seawater surrounding the macroalga. Estimation of photosynthetic carbon uptake and carbon loss was done as described by Kalokora et al. [20] using the following equations:

Photosynthetic carbon uptake = (DIC_initial_ − DIC_after light incubation_) − DIC_calcification_ (1)

Carbon loss = (DIC_initial_ − DIC_after equilibration_) − DIC_calcification_ (2)

### 4.3. Interrelation between Photosynthesis and Calcification

Downscaled and shortened setups were adopted for this experiment. Each macroalga with known and similar wet weight (5 g) was placed in 125 mL flasks filled with 140 mL of sterilized seawater. Measurements for each species and each treatment were based on five biological replicates (n = 5). 

This experiment was designed to compare calcification rates in the light to those obtained when photosynthetic light reactions were inhibited. First, we compared calcification rates in light and darkness. During the light exposure, an irradiance of approximately 150 μmol photons m^−2^ s^−1^ was provided for 1 h (Aquarium LED light A601, Chihiros, China). Calcification rates in the light were calculated from the TA measured before and after light exposure as previously described. The seawater was subsequently replaced, and macroalgae were maintained in fresh seawater in darkness for an additional 1 h. Calcification rates in the dark (Calcification_dark_) were calculated from the TA measured before and after incubation in darkness. Flasks filled with seawater without macroalgae were used as negative controls. Second, we compared calcification rates in the light and in the presence of 0.1 mM 3(3,4-dichlorophenyl)-1,1-dimethylurea (DCMU, the concentration was selected based on the preliminary test, data not shown). Calcification rates in the light were calculated from the TA measured before and after 1 h of light exposure as previously described. The seawater was subsequently replaced with seawater containing 0.5 mM DCMU, and irradiance was provided for an additional 1 h. DCMU inhibits photosynthetic electron transport from PSII to plastoquinone and is widely used as an inhibitor of photosynthesis [85]. The use of DCMU can determine whether the calcification and photosynthetic electron transport are interrelated [64,86]. Seawater prepared for the measurements of calcification rates in the light contained the same volume of ethanol used as the solvent in DCMU treatment (70 µL, corresponding to 0.05% *v*/*v*). Flasks filled with seawater without macroalgae were used as negative controls. Calcification rates in the presence of DCMU (Calcification_dcmu_) were calculated from the TA measured before and after incubation in the light. Seawater pH at the end of each step was also recorded. 

### 4.4. Statistical Analyses

All the analyses were performed using Statistica academic platform version 13.0 (StatSoft, Tulsa, OK, USA). Cochran’s test was used to verify the assumption of homogeneity of variance prior to conducting the analysis of variance (ANOVA). The statistical significance level was set at *p* < 0.05 in all analyses. 

Differences in %CaCO_3_ and the CaCO_3_ crystal morphology and densities among species were analyzed using one-way ANOVA. The difference in surface diameter of primary utricles (pU) diffusion pathway length was analyzed using a *t*-test. 

To characterize the effects of calcification and its effect on seawater chemistry, water chemistry and calcification rates were analyzed by repeated-measures ANOVA using the time of measurements as the within-group factor and macroalgal species as the categorical factor. Differences in each fate of carbon (carbon uptake by photosynthesis, carbon removal by calcification and carbon lost to the air) among species were analyzed using one-way ANOVA. Fisher’s least significant difference (LSD) test was used to compare these parameters across treatments. The relationships between calcification and carbon loss and between photosynthesis and calcification were analyzed by linear regression. 

Effects of inhibition of photosynthesis on seawater pH and calcification rates were tested by repeated-measures ANOVA using time of measurements as the within-group factor and macroalgal species as the categorical factor. Fisher’s LSD test was used to compare these parameters across treatments.

## Figures and Tables

**Table 1:** Morphological and anatomical characters of *Padina boryana*, *Halimeda opuntia* and *Halimeda macroloba*. Data are Mean ± SE. Different uppercase letters indicate statistical difference (Fisher’s LSD test, *p* < 0.05).

	*Padina boryana*	*Halimeda opuntia*	*Halimeda macroloba*
Specific thallus area (cm^2^ g^−1^ fw)	37.09 ± 1.83 ^a^	6.41 ± 0.28 ^b^	6.31 ± 0.40 ^b^
Specific thallus area (cm^2^ g^−1^ dw)	163.88 ± 13.37 ^a^	14.84 ± 1.32 ^b^	23.97 ± 1.50 ^b^
% Dry weight of calcium carbonate	46.89 ± 3.57 ^a^	81.44 ± 1.71 ^b^	77.35 ± 1.44 ^b^
Calcium carbonate polymorph	Aragonite	Aragonite	Aragonite
Site of calcium carbonate	Cell surface	Intercellular spaces	Intercellular spaces
Calcium carbonate crystal shape	Needle or rod shape	Needle or rod shape	Needle or rod shape
Crystal width (µm)	0.13 ± 0.02 ^a^	0.22 ± 0.02 ^b^	0.12 ± 0.02 ^a^
Crystal length (µm)	1.36 ± 0.05 ^a^	1.80 ± 0.09 ^b^	1.24 ± 0.05 ^a^
Crystal density (needles µm^−2^)	5.26 ± 0.41 ^a^	3.47 ± 0.12 ^b^	5.65 ± 0.34 ^c^
Surface diameter of primary utricle (µm)	-	23.67 ± 0.69 ^a^	28.31 ± 0.40 ^b^
Diffusion pathway length (µm)	-	5.95 ± 0.25 ^a^	22.11 ± 0.51 ^b^

^a, b, c^ Significant difference.

**Table 2:** A summary of seawater carbonate chemistry before an incubation with *Padina boryana*, *Halimeda opuntia* and *Halimeda macroloba* (initial), after 3 hours of incubation and after equilibration with air. The values presented were calculated from the average total alkalinity and pH (see Figure 2) using CO_2_ sys.xls program (version 2.3). Data are means ± SE (n = 9). Different uppercase letters indicate statistical difference (Fisher’s LSD test, *p* < 0.05).

**DIC**	*Padina boryana*		
	Initial	After 3 h Incubation	After Equilibration
CO_2_ (μM)	8.97 ± 0.22 ^a^	0.93 ± 0.26 ^b^	9.64 ± 0.51 ^a^
HCO_3_^−^ (μM)	1491.83 ± 6.64 ^a^	628.83 ± 93.61 ^b^	1438.83 ± 28.28 ^a^
CO_3_^2−^ (μM)	199.57 ± 3.80 ^a^	436.15 ± 20.38 ^b^	174.50 ± 6.66 ^c^
ΩCa	4.97 ± 0.09 ^a^	10.88 ± 0.50 ^b^	4.35 ± 0.16 ^a^
ΩAr	3.31 ± 0.06 ^a^	7.24 ± 0.33 ^b^	2.89 ± 0.11 ^c^
**DIC**	***Halimeda opuntia***		
	**Initial**	**After 3 h** **Incubation**	**After Equilibration**
CO_2_ (μM)	8.86 ± 0.10 ^a^	12.12 ± 1.41 ^c^	9.85 ± 0.56 ^a^
HCO_3_^−^ (μM)	1489.30 ± 6.19 ^a^	1281.85 ± 73.01 ^c^	1217.81 ± 71.85 ^c^
CO_3_^2−^ (μM)	200.56 ± 2.16 ^a^	114.93 ± 9.95 ^d^	125.61 ± 14.61 ^d^
ΩCa	5.00 ± 0.05 ^a^	2.86 ± 0.24 ^d^	3.13 ± 0.36 ^d^
ΩAr	3.33 ± 0.03 ^a^	1.90 ± 0.16 ^d^	2.08 ± 0.24 ^d^
**DIC**	***Halimeda macroloba***		
	**Initial**	**After 3 h** **Incubation**	**After Equilibration**
CO_2_ (μM)	8.71 ± 0.40 ^ad^	7.29 ± 0.81 ^d^	9.50 ± 0.25 ^a^
HCO_3_^−^ (μM)	1483.06 ± 4.73 ^a^	1210.38 ± 75.68 ^c^	1276.53 ± 53.11 ^c^
CO_3_^2−^ (μM)	203.04 ± 3.97 ^a^	168.60 ± 11.24 ^ac^	132.38 ± 10.27 ^d^
ΩCa	5.06 ± 0.09 ^a^	4.20 ± 0.28 ^c^	3.30 ± 0.25 ^d^
ΩAr	3.37 ± 0.06 ^a^	2.80 ± 0.18 ^c^	2.19 ± 0.17 ^c^

^a,b,c,d^ Significant difference.

## Data Availability

Request to corresponding author of this article.
